# Immunomodulatory Effect of Infectious Disease of a Breastfed Child on the Cellular Composition of Breast Milk

**DOI:** 10.3390/nu15173844

**Published:** 2023-09-03

**Authors:** Agata Tomaszewska, Alicja Jeleniewska, Klaudia Porębska, Katarzyna Królikowska, Agnieszka Rustecka, Agnieszka Lipińska-Opałka, Agata Będzichowska, Robert Zdanowski, Karolina Aleksandrowicz, Małgorzata Kloc, Bolesław Kalicki

**Affiliations:** 1 Department of Paediatrics, Nephrology and Allergology, Military Institute of Medicine—National Research Institute, Szaserów 128, 04-141 Warsaw, Poland; awesolowska1@wim.mil.pl (A.J.); kwisniewska1@wim.mil.pl (K.K.); arustecka@wim.mil.pl (A.R.); alipinska@wim.mil.pl (A.L.-O.); abedzichowska@wim.mil.pl (A.B.); kalicki@wim.mil.pl (B.K.); 2 Laboratory of Molecular Oncology and Innovative Therapies, Military Institute of Medicine—National Research Institute, Szaserów 128, 04-141 Warsaw, Poland; kporebska@wim.mil.pl (K.P.); rzdanowski@wim.mil.pl (R.Z.); kaleksandrowicz@wim.mil.pl (K.A.); 3 Transplant Immunology, The Houston Methodist Research Institute, Houston, TX 77030, USA; mkloc@houstonmethodist.org; 4 Department of Surgery, The Houston Methodist Hospital, Houston, TX 77030, USA; 5 Department of Genetics, MD Anderson Cancer Center, The University of Texas, Houston, TX 77030, USA

**Keywords:** breastfeeding, human milk, immunomodulation, cellular composition of milk, infection

## Abstract

Recent studies suggest that the content of immune components in milk is influenced by the mother’s health and also by the infant she feeds. We aimed to evaluate the effect of a child’s respiratory tract infection on the cellular composition of breast milk (neutrophils, monocytes, eosinophils, lymphocytes, and their subpopulations). Twenty-six breastfeeding mothers whose children were hospitalized for respiratory tract infections were enrolled in the study. The control group consisted of 23 mothers of healthy children. Regarding the children, baseline laboratory blood tests were performed, and nasal swabs were taken for the presence of RS virus. In the next step, milk samples were collected from the mothers to assess the cellular composition of the milk, including neutrophils, monocytes, eosinophils, lymphocytes, and their subpopulations. Significantly higher percentages of T lymphocytes (helper and cytotoxic lymphocytes) were observed in the milk of the studied mothers. There was a significantly higher percentage of milk lymphocytes in the group of affected children with confirmed RSV etiology than in children with excluded RSV etiology. A significant positive correlation was observed between the duration of infection and the percentage of milk NK cells and between milk CD19 lymphocytes and the child’s serum leukocytosis. This study may provide evidence of a link between cells in breast milk and disease in the breastfed infant. The severity of the infection, its duration, and the etiological agent of the infection may affect the cellular composition of milk.

## 1. Introduction

The World Health Organization (WHO) recommends exclusive breastfeeding for babies up to six months. Breastfeeding fully meets the infant’s needs for all essential nutrients, ensuring proper development during the first six months of life. As WHO experts emphasize, the promotion of natural feeding is one of the most effective strategies for improving population health.

Breastfed babies have been shown to be healthier than formula-fed babies. They have a reduced risk of infections (in particular, otitis media and gastroenteritis), necrotizing enterocolitis, cancers (lymphoblastic and myeloid leukemia), and metabolic diseases (including type 1 and type 2 diabetes) [1]. In addition, sudden infant death syndrome (SIDS) was significantly lower in breastfed than in formula-fed babies [2]. It has also been shown that breast suckling promotes the exercise of facial muscles, thus improving speech development [3].

Due to its unique composition and, as a result, preventive and health-promoting effects on the child’s body, breast milk is considered a so-called functional food. In addition to the nutritional values, it has many non-nutritional functions, including regulation of child development, protection against bacterial and viral infections, and functional maturation of the digestive tract [4].

The several health benefits associated with breastfeeding are driven by the combined action of the nutritional and bioactive components in human milk. In addition to nutritional function, breast milk is also immunoprotective. The leukocytes, antibodies, and immunomodulating factors (including lactoferrin, lysozyme, lactoperoxidase) present in the milk strengthen the infant’s immature immune system [5]. Therefore, breastfeeding should always be encouraged, not only for nutritional reasons but mainly because of its immunological properties.

Historically, breast milk leukocytes were thought to have little biological relevance for the infant because they could not survive the acidic environment of the stomach. However, the results of experimental studies in animal models suggest that leukocytes supplied with maternal milk migrate from the gastrointestinal tract into the blood of suckling and then translocate to the lymph nodes, liver, and spleen [6]. Now, we know that the main function of milk leukocytes is to support the infant’s active immunity through the phagocytosis of pathogens present in the infant’s body. Leukocytes also support the development of the infant’s immune system and modify the infant’s gastrointestinal microenvironment [7].

Among leukocytes, the most abundant are macrophages, which can phagocytose antigens without prior opsonization. In addition, they contain large amounts of secretory IgA antibodies that support the child’s immune system in fighting pathogens [8].

Lymphocytes are another leukocyte population in breast milk. The main fraction is T lymphocytes (about 80% of lymphocytes). Maternal T lymphocytes support functionally immature neonatal lymphocytes and promote their maturation [9]. B lymphocytes in the mammary gland differentiate into plasma cells producing IgA-class antibodies [10].

The composition of breast milk is dynamic and changes depending on the phase of feeding, time of day, or maturity of the infant. Colostrum, produced a few days after birth, has the highest leukocyte content. On the other hand, transitional and mature forms of milk have a low content of white blood cells (less than one-tenth of the leukocyte content of colostrum) [11]. The biologically active components of breast milk can be modified not only by internal factors but also by external factors [12]. The influence of antibiotic therapy [13], nicotinism [14], or maternal diet supplementation is well proven [15,16]. Some researchers believe that the immune composition of breast milk is even influenced by factors to which the woman was exposed during early childhood [17].

During a mother’s infection, the abundance of some immune cells in her milk increases significantly, which enhances the baby’s ability to protect itself against infection. Recent studies suggest that the content of immune components in milk is influenced not only by the mother’s health but also by the condition of the infant she feeds [18]. However, these reports are few and the results are inconclusive. Taking this into account, in the current study, we investigated whether an infection in a breastfed baby affects the immune system cells in breast milk.

## 2. Materials and Methods

### 2.1. Study Group

Twenty-six breastfeeding mothers whose children were hospitalized in the Department of Paediatrics, Nephrology, and Paediatric Allergology at the Military Institute of Medicine in Warsaw (Poland) between 2021 and 2022 for respiratory tract infections were enrolled in the study. 

Children who were exclusively breastfed were enrolled in the study. Partially breastfed children were excluded from the study.

Both mother and child were examined at the time of inclusion to the study. Women with symptoms of respiratory tract infection or chronic disease were excluded from the study. The child’s physical examination included information about the nature of the symptoms and their duration and the outpatient treatment used. Basic laboratory tests were performed on the blood, and nasal swabs were tested for RS virus. If pneumonia was suspected, a chest radiograph was performed. Table 1 shows the inclusion and exclusion criteria for the study.

In the next step, milk samples were collected from mothers to assess the cellular composition of the milk: neutrophils, monocytes, eosinophils, and lymphocytes (CD4+ T lymphocytes, CD8+ T lymphocytes, NK cells, B lymphocytes).

The control group consisted of 23 mothers breastfeeding infants aged 1–6 months. Both mother and child were healthy at the time of inclusion in the study.

### 2.2. Milk Sampling

Milk was collected on the first day of hospitalization, at least 2 h after the last feeding, from the breast that the baby was fed (to eliminate variability in milk composition during feeding). Breast pumping was always performed under the same conditions—in a specially prepared room for the parents, providing an intimate atmosphere, using an electric breast pump (model Symphony, Medela; Baar, Switzerland). Samples were collected in sterile, disposable 20 mL containers. They were then transported in an isothermal bag to the Laboratory of Oncology and Molecular Biology of the Military Institute of Medicine.

### 2.3. Flow Cytometry of Breast Milk

The study used proprietary methodology developed on the basis of previous experience [19] and modified for the purposes of milk testing [20,21].

For extracellular staining, DPBS 10X (Corning), 4% PFA (Merck), and appropriate antibody-fluorochrome tandems (Beckman Coulter) were used. To determine the general phenotype of leukocytes, the Leukocyte Common Antigen: anti-CD45-KRO was used. For the characteristics of individual subpopulations among leukocytes (CD45+ cells), the following antibodies were applied: anti-CD3-FITC, anti-CD8-PC5, anti-CD4-APC for T lymphocytes, anti-CD19-PC 7 for B lymphocytes, and anti-CD (16 + 56)-PE for NK cells.

Cells CD45 + CD14+ were qualified for the macrophage/monocyte population. CD45 + CD16- with adequate SSC position cells were qualified to neutrophils. Eosinophil populations were marked as CD45+ cells with the brightest autofluorescence signal in empty UV channel. 

In the study material, cells with a stem cell phenotype were characterized based on the following markers: CD105-PC7, CD34-ECD, CD73-PE, CD45-AF700, and CD44-AF750. For epithelial cells, the anti-CD326-APC antibody was used.

Next, 20 mL of cold breast milk and 20 mL of cold 1X PBS were added to a falcon tube and centrifuged at 1000 RCF for 20 min at 4 °C. The pellets were rinsed and recentrifuged twice. Pellets from the last centrifugation were resuspended in 1X PBS. Then, 100 µL samples were dispensed into panels (phenotype, stem cells) and incubated for 30 min in the dark at room temperature (RT). After labeling, cells were fixed with 4% PFA and washed with 1X PBS. Samples labeled by all panels were resuspended in 1X PBS. Stained samples were assayed on a Cytoflex, Beckman Coulter flow cytometer, and in FCAP Array software (CytExpert, Version 2.3.0.84).

### 2.4. Statistical Analysis

The results were statistically analyzed using the StatSoft software (STATISTICA 2014). The analyses were initially verified using the normal distribution diagrams (Smirnov and Liliefors test). The Student’s *T*-test was used to evaluate variables with normal distribution. For variables inconsistent with the normal distribution, non-parametric tests were used (U-Mann–Whitney test). Correlation was calculated using Spearman test (variables lacking the normal distribution) or Pearson’s correlation factor (variables with normal distribution). A *p*-value < 0.05 was considered statistically significant.

## 3. Results

### 3.1. Characteristics of the Study Group

(a) Mothers

Primiparous women accounted for 4% of all mothers studied. Thus, 13/26 women (50%) declared the presence of chronic diseases during pregnancy. In 17/26, there was a need for pharmacotherapy (65%). The mean duration of pregnancy was 39 weeks (±1 week). The predominant route of delivery was natural childbirth (17/26).

(b) Infants

Mean birth weight and length were 3566 g (±303 g) and 55 cm (±2 cm), respectively. Most infants (24/26) scored 10 on the Apgar scale.

(c) Infection

The mean duration of illness symptoms before admission was 5 ± 6 days. Cough was the predominant symptom, present in 24/26 (92%) infants. Rhinitis was present in 20/26 (77%) infants. Dyspnea and fever were present in 7/26 (27%) and 6/26 (23%) children, respectively. Most children (24/26, 92%) were treated symptomatically before hospitalization; 2/26 received antibiotic therapy before hospitalization (in both cases—clarithromycin).

The mean duration of hospitalization was 6 ± 2 days. All children were diagnosed with pneumonia. In 12/26 cases, the cause of the lower respiratory tract infection was RS virus. In 20/26 children (77%), antibiotic therapy was administered during hospitalization.

Table 2 shows a comparison between the study and the control group. Most characteristics were not statistically different, except significantly fewer primiparous women in the study group than in the control group.

### 3.2. Influence of Infection on the Immunological Composition of Milk

Significantly higher percentages of T lymphocytes (CD3 marker)—both helper T lymphocytes (CD4 marker) and cytotoxic T lymphocytes (CD8 marker)—were observed in the milk of mothers in the study group (Table 3).

**Figure 1:**
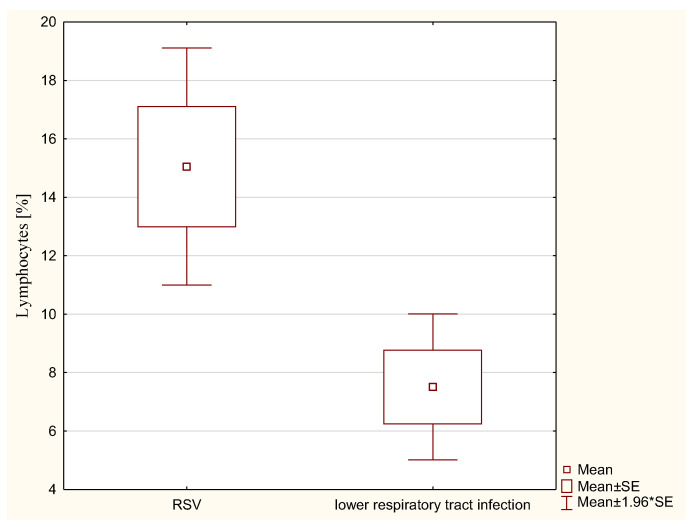
Percentage values of lymphocytes present in breast milk in children with RSV (+) and RSV (−) infection.

In addition, children with RSV had a significantly higher percentage of milk lymphocytes than children without RSV infection (15.03% ± 6.55 vs. 7.51% ± 4.41; *p* < 0.05) (Figure 1).

**Figure 2:**
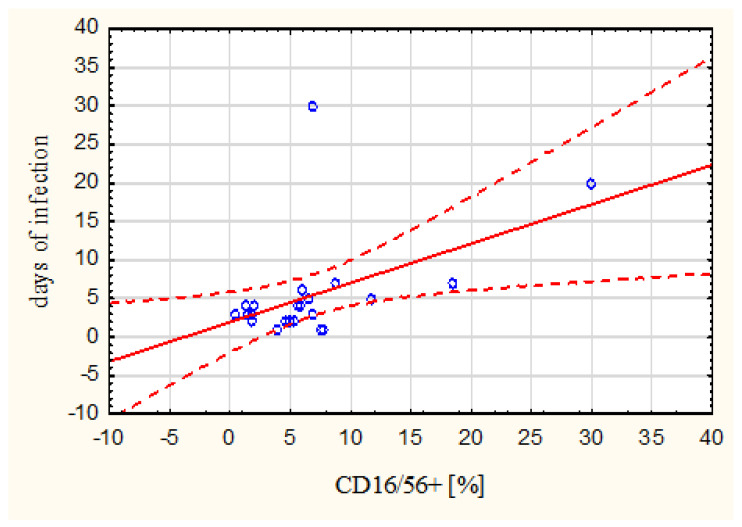
The relationship between CD16/56 percentage value and duration of infection (*Points on the graph—individual analyzed cases, Solid line—trend line, dashed line—95% confidence curve)*.

A significant positive correlation was observed between the duration of infection and the percentage of CD16/56 in the milk (Spearman’s correlation coefficient r = 0.49) (Figure 2). The longer the infection lasted, the more CD15/56 cells were observed in breast milk.

A positive correlation was observed between the immune cells in milk and the markers of inflammation—the higher the leukocytosis in the child’s blood, the higher the CD19 lymphocyte percentage in the mother’s milk (Table 4).

## 4. Discussion

In addition to its nutritional values, breast milk has a number of other functions, of which defense and protection are among the most important. The cells of the immune system in breast milk provide the child with both a passive (transfer of antibodies) and an active immune response (active phagocytosis of pathogens) [7]. The benefits of breast milk make it one of the most important factors in protecting infants against the morbidity and mortality of infectious diseases [22]. Numerous factors within human breast milk act in a complementary fashion to protect against infection. 

Most previous research on the immune cells in breast milk has focused on colostrum, while very little is known about immune cells in mature milk. It is also not fully known whether infection in the breastfed infant is a factor affecting the cellularity of breast milk. We assessed the effect of respiratory tract infection in a breastfed infant on the cellular composition of mature milk. In order to eliminate the potential impact of maternal infection on the immunological composition of milk, only healthy mothers were included in the study. 

The following hypothesis was adopted in this study: a respiratory infection in an infant increases the amount of immune system cells in breast milk. We observed higher values of T lymphocytes (marker CD3), helper T cells (marker CD4), and cytotoxic T cells (marker CD8) in the milk of mothers in the study group compared to the milk of mothers in the control group. It can be speculated that the increased number of cells in breast milk may help the nursing baby cope with the infection. This is confirmed by the proven ability of macrophages and leukocytes in human milk to kill enteropathogenic Escherichia coli, Giardia lamblia, Staphylococcus aureus, and Candida albicans [23].

The mechanism underlying this phenomenon is unclear and requires further study. There is a hypothesis that during breastfeeding, a phenomenon called ‘backwash’ occurs. After the contraction of the tubules, leading to the outflow of milk, there is a backflow of a small amount of the infant’s oral contents into the mammary gland [24]. Studies have shown that infant saliva reacts with breast milk to form a combination of biochemical metabolites that induces fundamental changes in milk composition [25]. It could also be possible that microorganisms from the infant could be transferred back into the breast, most likely during a pause in suckling, stimulating a local immune response [26].

Another explanation is that an infant’s respiratory infection actually infects the mother as well, causing an inflammatory reaction in her body that causes an increased secretion of white blood cells into her milk. It can be speculated that the inflammatory response may increase the number of leukocytes in the blood or attract more cells to the mammary gland, causing an increase in the number of cells secreted in breast milk. Exposure of the mother to the infant’s infection may stimulate an immunological response in the mother that is manifested without evident symptomatology but which influences breastmilk leukocyte content. 

Lymphocytes are not the most abundant leukocyte population present in breast milk. However, as shown in our study, their number can increase following infection in a breastfed infant. Lymphocytes transferred with breast milk perform their functions not only in the infant’s gastrointestinal tract but also in the distant tissues, which they enter from systemic circulation [27,28]. It is, therefore, possible to hypothesize that milk-derived T cells and their products provide direct immunological help to the infant’s immature immune system and even transfer immunological information to the newborn’s immune cells.

We still do not know why the infant immune system does not fight immune cells entering from the circulation. It is probably related to the phenomenon of the ‘breastfeeding-induced maternal microchimerism’. This process has been observed in experimental studies on animal models. Maternal milk cells have been shown to nest in the tissues of the offspring, which probably induces tolerance to maternal antigens [29].

Previous research on immune cells in human milk focused on the impact of maternal infection on the lactation process. There are few publications assessing the effect of infant’s infection on the cellular composition of breast milk. Hassioutu et al. [30] assessed the impact of maternal and/or child infection on milk composition. They observed a significant increase in milk leukocytes in both the mother’s infection and the infant she fed. After recovery, the white blood cell count returned to baseline values. Furthermore, exclusive breastfeeding was associated with higher milk leukocyte values. Similar observations were made by Riskin et al. [31] in a study involving a group of 31 breastfed infants with infection. They observed an increase in the total number of leukocytes (mainly macrophages) and the cytokine TNF-alpha.

Children with fever—caused by respiratory tract infection, gastrointestinal tract infection, or genitourinary tract infection—were eligible for the above-mentioned studies. Only children with lower respiratory tract infections were analyzed in our study. Therefore, caution should be exercised in comparing our results with those cited above. In addition, none of the above-mentioned studies extracted the lymphocyte population from the total leukocyte count. The authors of the present study attempted to assess the impact of a child’s infection on specific leukocyte subpopulations—neutrophils, eosinophils, monocytes, and lymphocytes, along with subpopulations.

Our analysis showed that the influence of infant infection on the cellular composition of breast milk has a multifaceted dimension and also depends on the type of infection and its severity. The breastmilk of mothers whose infants were confirmed to be infected with RSV was characterized by a higher percentage of lymphocytes compared to infants in whom this etiology of infection was excluded. The effect of RS virus infection on the cellular composition of breast milk was also observed by Bryan et al. [32]. Children with bronchiolitis of RSV etiology were eligible for the study. They observed significantly higher leukocyte values in milk compared to healthy children. However, there was no difference in the proportions of the milk leukocyte population.

In the study cited above, the control group consisted of healthy children. In contrast, the reference point in our study was infants with respiratory tract infections of an etiology other than RSV. The results of the self-analysis suggest a different response of the maternal immune system to infection of the breastfed infant in the case of RS virus infection. There are no data available in the literature that directly address this issue. The immunopathology of RS virus infection is mainly related to its effect on dendritic cells, leading to an increased influx of lymphocytes and an enhanced immune response [33]. Extrapolating, it can be assumed that similar processes also occur in the mammary gland. Perhaps sick infants take in smaller volumes and more frequently, which may change the process of cell accumulation in the breast. However, in a study by Bryan et al. [32], there was no difference in the volume of milk obtained from full pumps in the sick and healthy groups, indicating a small effect of the infant’s disease on the total volume of milk.

The results of our study indicate that the cellular composition of breast milk also depends on the severity of the child’s infection. More B lymphocytes (marker CD19) were in the milk of mothers whose children had higher blood leukocytosis. In addition, an increase in the content of NK cells (marker CD16/56) in milk was observed with the duration of the infection. The results of our own study may confirm the dynamic nature of the immune defense provided to sick breastfed infants. Although B lymphocytes and NK cells make up only a few percent of milk lymphocytes (6% and 2%, respectively) [34], they are crucial for fighting pathogens. In the case of NK cells, this is through antibody-dependent cellular cytotoxicity (ADCC) and antibody-dependent cellular phagocytosis (ADCP). However, little is known, still, about the role of milk NK cells in combating pathogens in the child. The function of B lymphocytes is much better understood. It has been shown that in the mammary gland, they undergo terminal differentiation into plasma cells that produce antibodies in the IgA, IgG, or IgM class [35]. The type, structure, and concentration of these antibodies differ from those found in plasma [36]. The predominant Ig in human milk is secretory IgA (sIgA), which can neutralize pathogens before they encounter epithelial cells. SIgA is resistant to digestive enzymes in the infant’s gastrointestinal tract, is not absorbed, and can, therefore, act locally on the gastrointestinal tract to protect the infant from infection [37].

Although the current study provided new information on the immunomodulatory effect of infectious diseases in children on milk composition, it had some limitations. The main limitation was the small sample size. This was due to the difficulty in qualifying exclusively breastfeeding mothers. Any supplementary feeding with modified milk (also in the neonatal unit) was an exclusion criterion from the study. Epidemiological studies conducted in Poland indicate a small proportion (about 6%) of women exclusively breastfeeding in the first six months of their child’s life [38]. We intend to perform an analysis comparing the effects of exclusive and partial breastfeeding on the composition of breast milk. There are reports that exclusive breastfeeding is associated with higher baseline leukocyte levels in breast milk under healthy conditions [39].

Another limitation was the lack of determination of cytokines and immunoglobulins in milk. Also, examination of various subpopulations of lymphocytes in the study was impossible for technical reasons (limited number of patients and research material). Research will continue, and the role of individual cell subpopulations will be studied in detail. 

In this study, only the effect of a child’s infection on the composition of milk was assessed. An analysis of the influence of maternal factors on the composition of milk is planned.

Our findings support a dynamic immune relationship between a breastfeeding mother and her infants. Formula does not offer this protection and the ability to adjust to infant needs. Thus, these findings present new information that is relevant to updating public policy on early infant nutrition that maximizes immunological development and protection against infections.

In summary, the results of our study indicate a multifaceted effect of infectious disease in the child on breast milk composition. This study may provide evidence of a link between cells in breast milk and disease in the breastfed infant. Both the duration and severity of the infection and the etiological agent may contribute to changes in the content of immune cells in breast milk. Further research is needed to elucidate the immune mechanisms underlying these responses as well as their clinical relevance.

## Figures and Tables

**Table 1:** Inclusion and exclusion criteria.

Inclusion Criteria	Exclusion Criteria
Exclusive breastfeeding; Age of child 1–6 months; No chronic diseases in the child; Symptoms of respiratory tract infection in the child; No symptoms of respiratory tract infection in the mother.	Partial breastfeeding; Supplementary feeding with modified milk; Bottle feeding of breast milk; Symptoms of respiratory tract infection in the mother; Chronic disease in the mother; Current pharmacotherapy in the mother; Child’s age < 1 month or >6 months.

**Table 2:** Study and control group characteristics.

	**Mothers**		
**Variable**	**Study Group (n = 26)**	**Control Group (n = 23)**	***p***
Age (years)	33 ± 3	32 ± 3	ns.
Firstborn (n, %)	1 (4%)	11 (48%)	<0.05
Duration of pregnancy (weeks)	39 ± 1	40 ± 1	ns.
	**Infants**		
**Variable**	**Study Group (n = 26)**	**Control Group (n = 23)**	***p***
Weight (g)	3566 ± 303	3735 ± 510	ns.
Length (cm)	55 ± 2	56 ± 3	ns.
CRP [mg/dL]	1.47 ± 2.16	-	-
WBC [×10^9^/L]	13.08 ± 5.02	-	-
Lymphocytes	6.78 ± 2.89	-	-
Lymphocytes [%]	54.19 ± 18.04	-	-
Neutrophils	3.96 ± 3.38	-	-
Neutrophils [%]	29.99 ± 17.99	-	-
ERC [mm/h]	19.63 ± 16.05	-	-

ns.—statistical non-variables; n—Group size; CRP—C-reactive protein, WBC—white blood cells, ERC—erythrocyte sedimentation rate.

**Table 3:** Immunological composition of milk in the test group and in the control group.

Variable	Study Group (n = 26)	Control Group (n = 23)	*p*
CD8+ [%]	27.09 ± 14.15	16.32 ± 10.27	0.005 *
CD4+ [%]	32.72 ± 14.85	22.58 ± 11.35	0.012 *
CD3+ [%]	86.97 (77.09–91.80)	75.57 (63.20–83.75)	0.006 *
CD19+ [%]	3.62 (2.35–6.92)	4.81 (2.82–7.19)	ns.
CD16/56 [%]	5.96 (2.91–8.21)	9.22 (4.85–14.12)	ns.
CD45+ [%]	4.23 (3.21–8.00)	5.37 (4.56–8.29)	ns.
Neutrophils [%]	10.28 (5.18–22.99)	7.00 (3.19–21.24)	ns.
Monocytes [%]	34.74 (19.59–44.94)	31.06 (15.22–42.55)	ns.
Eosinophils [%]	0.67 (0.38–1.39)	0.78 (0.45–1.62)	ns.

*—statistical significance; n—Group size.

**Table 4:** Correlation ratio between cells present in milk and infant inflammatory markers.

Variable	Correlation Ratio	
	CRP	WBC
CD8+	−0.176641	−0.317352
CD4+	0.064060	−0.154985
CD3+	0.168421	−0.233185
CD19+	0.077621	0.424169 *
CD16/56+	−0.174016	0.191124
CD45+	−0.154986	0.239003
Neutrophils	−0.188595	0.119661
Monocytes	0.326095	0.330613
Eosinophils	0.211600	−0.042598

*—statistical significance, WBC—white blood cells.

## Data Availability

The datasets used and/or analyzed during the current study are available from the corresponding author on reasonable request.
